# Copper and zinc deficiency to the risk of preterm labor in pregnant women: a case-control study

**DOI:** 10.1186/s12884-023-05625-2

**Published:** 2023-05-19

**Authors:** Haniyeh Gohari, Nasim Khajavian, Azamsadat Mahmoudian, Roghaieh Rahmani Bilandi

**Affiliations:** 1 Department of Midwifery, Gonabad Branch, Islamic Azad University, Gonabad, KHorasan Razavi, Iran; 2 Department of Biostatistics, Social Development and Health Promotion Research Center, Gonabad University of Medical Sciences, Gonabad, Khorasan Razavi, Iran; 3 Department of Obstetrics and Gynecology, School of Medicine, Allameh Bohlool Gonabadi Hospital, Khorasan Razavi, Iran; 4 Department of Midwifery, Social Development and Health Promotion Research Center, Faculty of Medicine, Gonabad University of Medical Sciences, Gonabad, Khorasan Razavi, Iran

**Keywords:** Antioxidant, Micronutrient, Copper, Zinc, Preterm delivery

## Abstract

**Objective:**

The present study explored the relationship between maternal copper and zinc levels and preterm labor.

**Design:**

The design of the present study was a case-control. Two groups were matched in terms of early-pregnancy body mass index (BMI), pregnancy and childbirth rating, education level, income, and employment status. Blood samples were taken from mothers after meeting the inclusion criteria when admitted to the maternity ward to check copper and zinc serum levels. Demographic and midwifery data were also collected using a questionnaire and patient records. The data were analyzed in SPSS26 using independent-samples T-test, chi-square, Fisher exact test, and regression analysis, and the *p* < 0.05 was considered statistically significant.

**Setting:**

Bohloul Hospital in Gonabad, Iran.

**Participants:**

The subjects were 86 pregnant women visiting the hospital in two cases (preterm delivery) and control (term delivery) groups.

**Results:**

The mean serum level of zinc in the case group (preterm delivery) (44.97 ± 13.06 µg/dl) was significantly lower than the control group (term) (52.63 ± 21.51 µg/dl), and the mean serum level of copper in the case group (149.82 ± 53.13 µg/dl) was significantly lower than the control group (183.97 ± 71.40 µg/dl).

**Conclusion:**

As the findings showed, copper and zinc serum levels in mothers with preterm delivery were significantly lower than mothers with term delivery, which shows the biological role of these elements in the pathogenesis of preterm delivery.

## Introduction

Preterm delivery is a primary issue discussed in the medical profession and health, leading to lifetime disability for humans [1]. WHO defined preterm birth as any birth before 37 completed weeks of gestation, or fewer than 259 days since the first day of the woman’s last menstrual period (LMP) [2]. The prevalence of preterm delivery varies widely among countries through the world [3] and it has prevailed despite many advances in medicine [4]. WHO estimated the worldwide prevalence of preterm delivery at approximately 15 million annually, and a study in the United States found that one in ten babies was born too early [5].

Approximately two-third of neonatal mortalities within the first year of life are due to preterm birth, and prematurity is also the second leading cause of mortality in children younger than five years [6]. The causes of preterm delivery can vary, with different features often interacting with each other, and this complexity makes efforts extremely hard to prevent and treat this complication. This issue is particularly relevant in preterm delivery rupture of membranes and spontaneous preterm labor, which together account for 70–80% of preterm deliveries [7].

Preterm delivery is well documented to be associated with oxidative stress so that women who experience preterm delivery exhibit higher levels of oxidative stress in the body [8]. Oxidative stress is induced by an imbalance between free radicals and the antioxidant defense system [9]. According to the existing body of research, oxidative stress causes preterm delivery by reducing cellular defense and damage to the collagen in the amniotic cells of the embryonic membrane [10].

Micronutrients, such as zinc and copper, are involved in synthesizing DNA, RNA and collagen and play a key role in reducing peroxidation and oxidative stress [11, 12]. Zinc acts as an antioxidant by stabilizing the membrane structure, protecting the sulfhydryl groups in proteins, and over-regulating the expression of a metal-containing protein named metallothionein. In addition, zinc suppresses anti-inflammatory responses. Otherwise, it can increase oxidative stress [13, 14]. Zinc is particularly important during pregnancy, and the need for zinc increases during this period. Plasma zinc levels are reduced during pregnancy, which affects both fetal and maternal health [7, 15].

Copper is also an active oxidation metal that reduces oxidation by catalyzing hydroxyl radicals [16]. It also plays an important role in maternal and fetal health. The fetus depends on copper in the mother to provide its required copper, and approximately three-quarters of fetal demand for copper are supplied by the mother’s copper during the last months of pregnancy [17].

The high prevalence of preterm delivery in populations with low socioeconomic status confirms the role of nutrition, particularly micronutrients, in the etiopathogenesis of preterm delivery [18, 19]. Several studies indicated that deficiency in copper and zinc during pregnancy is associated with low birth weight (LBW) and preterm delivery; however, this is not confirmed by all the existing studies. This study can help identify the pathogenesis of preterm delivery in countries marked by micronutrient deficiencies, particularly in Asian countries [20]. Due to the deficiency of these micronutrients during pregnancy and the increasing need of pregnant women for these micronutrients, the present study can be useful in providing prenatal care and supplementation during pregnancy. Accordingly, the present study was conducted to compare the serum levels of copper and zinc in women with preterm and term delivery.

## Methodology

### Research population and design

**Fig. 1:**
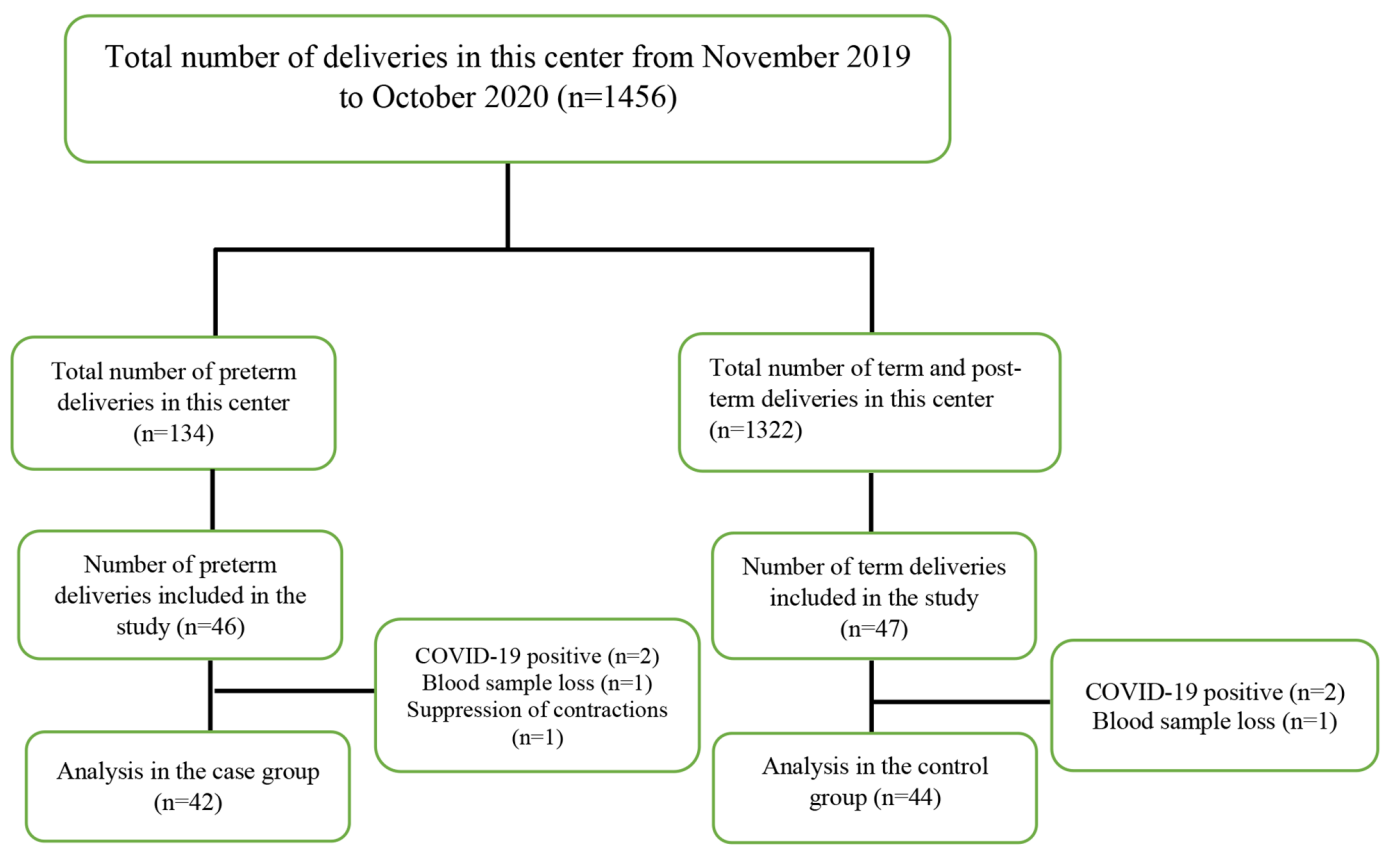
Flowchart of participation in the two study groups

Having received an approval by the ethics committee approval (#IR.GMU.REC.1398.120) the present case-control study was conducted from November 2019 to October 2020 in Allameh Bohloul Hospital in Gonabad, Iran. The research population consisted of all pregnant women with symptoms of preterm delivery and normal pregnant women who visited the maternity ward. The sample size was estimated at 42 using the commonly used sample size formula in the literature [21]. With a possible attrition rate of 5%, 46 subjects were assigned to each research group. The inclusion criteria were the absence of cases with multifetal pregnancies, physical and psychological medical diseases in mothers, history of habitual abortion, uterine anomaly, uterine fibroids (also called leiomyomas), pregnancy with an IUD, history of preterm delivery, cerclage, abruption, Previa, intrauterine growth restriction, smoking, alcohol and drug abuse, polyhydramnios, oligohydramnios, urinary tract infection, and vaginal infection at any time during pregnancy. In addition, women suspected of or infected with COVID-19 were excluded from the study. Mothers were supposed to have two or more regular uterine contractions at 45–90 s intervals every 10 min, accompanied by changes in the cervix. Contractions were measured manually and also with a tocodynamometer monitoring device. Exclusion criteria were unwillingness to take blood samples, loss of blood samples, and mothers receiving tocolytics to suppress contractions and not having a preterm delivery. The participation flowchart is presented in Fig. 1. The participants’ gestational age was 26-36.6 weeks in the case group (preterm delivery), and 37–40 weeks in the control group (term). The gestational age was estimated based on the first day of the last menstrual period or the first-trimester ultrasound, and the ultrasound criterion was accepted with a difference of more than ten days. Mothers in both groups were matched for pregnancy and childbirth rating, BMI at pregnancy, education level, income, and employment status.

### Data and sample collection

A researcher was present in the obstetrics and gynecology ward of Allameh Bohloul Hospital, Iran for the whole sampling process which took 11 months. After visiting the pregnant mothers with symptoms of preterm delivery and a definitive diagnosis of preterm delivery by a gynecologist and based on the inclusion criteria, the sampling was done. The objectives of study were explained to the participants. They were provided with all the information needed, and a written informed consent was obtained from them all. A non-probability sampling was performed through a convenience method. The data on midwifery and demographic variables were collected in face-to-face interviews with mothers and hospitalization records were also used at the time of sampling. A blood sample with a 5 ml syringe, preferably from the antecubital vein, was taken from mothers at the time of admission to hospital and before any medical treatment. This sample was collected in a clot test tube. All samples were washed with water and deionized acid to be free of trace elements. Whole blood was used to isolate serum using a centrifuge device with 3000 rpm for 5 min, and the resulting serum was carefully collected in well-rinsed acid-sealed polyethylene tubes and stored at -20 °C for later analysis. Then, serum levels of copper and zinc were measured using a Randox kit (Miindry BS-600, Germany), which has a global standard and was calibrated daily by the relevant expert. In addition, the samples were measured by an experienced individual to reduce human error. Data analysis was done in SPSS26 and independent-samples T-test, chi-square, Fisher exact test, and regression analysis. The *p*-value < 0.05 was considered as statistically significant.

## Results

According to the inclusion criteria, finally, 46 mothers in the case group (preterm delivery) and 47 in the control group (term) were included in the study; however, four women in the case group (preterm delivery) and three in the term group were excluded due to blood sample losses, positive COVID-19 tests, and suppression of uterine contractions. The mean age of mothers in the case group and in the control group were 28 ± 6 and 26 ± 6 years, respectively, and the two groups were homogeneous in terms of age. Moreover, the two groups were homogeneous in terms of pregnancy and childbirth rating, history of abortion, BMI, and demographic variables, such as mothers’ and spouses’ education level, mothers’ and spouses’ employment status, and income levels. This information is summarized in Table 1.

**Table 1:** Distribution of demographic variables and pregnancy characteristics

Variable		Case Group No. (%) n = 42	Control Group No. (%) n = 44	Significance Level
Pregnancy Rating*	1	17 (40.5)	20 (45.5)	X^2^ = 0.46 P = 0.9
	2	13 (31.0)	14 (31.8)	
	≥ 3	12 (28.5)	10 (22.7)	
BMI*	< 18.5 (Weight Loss)	5 (11.9)	1 (2.27)	X^2^ = 3.3 P = 0.2
	18.5–24.9 (Normal)	32 (76.2)	38 (86.36)	
	25-29.9 (Overweight)	5 (11.9)	5 (11.37)	
Occupation**	Household	37 (88.1)	39 (88.6)	*Fisher Exact Test* = 2.8 P = 0.99
	Home Jobs	1 (2.4)	1 (2.3)	
	Employee	3 (7.1)	3 (6.8)	
	Student	0 (0.0)	1 (2.3)	
	Self-employed	1 (2.4)	0 (0.0)	
Mother’s Education**	Illiterate	1 (2.3)	1 (2.3)	*Fisher Exact Test* = 1.7 P = 0.69
	High school	13 (31.0)	18 (40.9)	
	Diploma	17 (40.5)	18 (40.9)	
	University	11 (26.2)	7 (15.9)	
Income Level**	Less than enough	4 (9.5)	4 (9.1)	*Fisher Exact Test* = 0.4 P = 0.8
	Enough for Living Expenses	36 (85.7)	39 (88.6)	
	More than enough	2 (4.8)	1 (2.3)	

* Chi-square test, ** Fisher exact test

The mean serum content of zinc was 44.97 ± 13.06 µg/dl in the case group (preterm delivery) and 52.63 ± 21.51 µg/dl in the control group (term). The results of the independent-samples *T*-test indicated a statistically significant difference between the two groups for the mean serum level of zinc (*p* = 0.049; Table 2).

**Table 2:** Mean (M) and standard deviation (SD) of zinc serum (µg/dl) in case and control groups

Variable	Preterm Delivery Group (M ± SD)	Term Group (M ± SD)	Independent T-test Result
Serum zinc (µg/dl)	44.97 ± 13.06	52.63 ± 21.51	T = 2.0 p-value = 0.049

The mean serum content of copper was 149.82 ± 53.13 µg/dl in the case group (preterm delivery) and 183.97 ± 71.40 µg/dl in the control group (term). The results of the independent-samples *T*-test indicated a statistically significant difference between the two groups for the copper mean serum (*p* = 0.01; Table 3).

**Table 3:** Mean (M) and standard deviation (SD) of serum copper (µg/dl) in case and control groups

Variable	Preterm Delivery Group (M ± SD)	Term Group (M ± SD)	Independent T-test Result
Serum copper (µg/dl)	149.82 ± 53.13	183.97 ± 71.40	T = 2.5 p-value = 0.01

The logistic regression analysis, univariate and multivariate with adjustment of confounding factors and the average confidence interval of 95% were used. In univariate analysis, in which each variables enter the model alone, the results showed that with every one unit of increase in copper, the odds of preterm delivery increase (OR = 1.009 and P = 0.019). The zinc variable along with the copper variable at a significance level of *P* < 0.2 entered the multiple logistic regression model. The results of multiple logistic regression showed that after adjusting the effect of these variables, there was a statistically significant relationship between copper and preterm delivery (*P* = 0.025); however, the zinc variable in the regression model was not statistically significant (*P* < 0.05; Table 4).

**Table 4:** Results of univariate and multivariate logistic regression analysis

Logistic Regression								
Regression	**Simple**				**Multiple**			
Variable	**Odds Ratio**	**Confidence Interval 95%**		**Significance Level**	**Odds Ratio**	**Confidence Interval 95%**		**Significance Level**
		**Lower**	**Upper**			**Lower**	**Upper**	
Zinc	1.029	0.998	1.061	0.063	1.027	0.996	1.059	0.092
Copper	1.009	1.001	1.017	0.019	1.008	1.001	1.016	0.025

## Discussion

Zinc and copper as two essential trace elements, have different biochemical functions in humans; therefore, their deficiency can affect the body differently [22]. The deficiency of these elements is common in developed countries; however, it is one of the most important health issues in developing countries due to poor nutritional status and high requirements [23].

The results of the present study which compared the serum levels of copper and zinc in mothers with preterm delivery and women with term delivery indicated a significant relationship between low levels of copper and zinc in mothers with preterm delivery, as addressed in the following.

Maamouri et al. (2012) studied the relationship between maternal serum levels of zinc and preterm delivery and found that low maternal serum zinc levels were associated with preterm delivery. This is consistent with the present study by comparing the serum levels of zinc in mothers with term and preterm deliveries [21]. A systematic review by Mori et al. (2015) on the effect of zinc supplementation on pregnancy outcomes showed that taking 15–44 mg of zinc supplementation per day is associated with a 14% reduction in preterm birth, which may indicate a reduction in maternal serum zinc, resulting in better pregnancy outcome with zinc supplementation [24]. Wang et al. (2019), in a retrospective cohort study, reported that mothers’ serum zinc concentrations during pregnancy were negatively associated with the risk of preterm delivery in the Chinese population [25]. According to a systematic and meta-analysis study conducted in 2016 on the relationship between zinc in maternal diet and adverse pregnancy outcomes, 16 studies found no relationship between maternal zinc nutritional status and spontaneous preterm delivery [26]. In addition, according to the results of a case-control study by Chiudzu et al. (2020), no relationship was found between high levels of mothers’ serum zinc and spontaneous preterm delivery, which is completely in contrast to the present findings [27]. Demirtürk (2006), in a case-control study, found no statistically significant relationship between preterm delivery and the level of mothers’ serum zinc [28].

The contradictory findings of these studies can be attributed to racial differences and discrepancies in social and economic status in different communities. As Mori et al. [24] contended, differences in research findings may be due to differences in the study setting, as zinc deficiency is more prevalent in developing countries than in developed countries. Such a phenomenon is likely to be related to low social status [24]. Another reason for the discrepancy of findings is related to the research methodology. The studies by Chiudzu [27] and Demirtürk [28] are relevant case-control studies. The strength of the present study compared to the aforementioned studies is the matching of participants in terms of the main variables. This finding was related to the effect of zinc serum on pregnant women with preterm delivery, in which the deficiency in zinc level leads to estrogen dysfunction, in turn leading to uterine muscle contraction, cervical dilatation, and premature rupture of the amniotic sac [29]. The zinc has also been indicated to be involved in the synthesis of prostaglandins and collagen; therefore, its absence may lead to premature rupture of the amniotic sac [30]. Zinc supplementation has shown to reduce the risk of preterm delivery by lowering the incidence or severity of maternal infections, which is a risk factor for preterm birth [31].

The results of the present study on comparing the serum copper levels of term and preterm mothers showed that the copper mean serum of preterm mothers was significantly lower than term mothers. Vukelić et al. (2012) conducted a case-control study to determine the relationship between copper serum and pregnancy consequences. The results indicated that low serum levels of copper are associated with a risk of preterm delivery [32]. Furthermore, the results of Demirtürk’s case-control study are consistent with the results of the present investigation [28].

In contrast, some studies did not show a significant relationship between mothers’ serum copper and preterm delivery [28, 33, 34]. The strength of the present study compared to others is that a large number of interfering factors in preterm delivery were eliminated by applying the inclusion criteria, and their impact was controlled. This study also compared copper and zinc levels in mothers with spontaneous preterm delivery, while some studies assessed preterm delivery for other reasons. It is noteworthy that Liu et al. (2019), in contrast to the above-mentioned studies, reported that an increase in serum levels of copper in the first half of pregnancy had consequences for preterm delivery and challenged the findings of other studies [35]. It is also discussed whether a decrease or increase in copper can lead to spontaneous preterm delivery. Moreover, the serum level of copper in which trimester of pregnancy has a more prominent role in the occurrence of preterm delivery requires more longitudinal studies. The contradictory findings in the literature can be due to different research methodologies of measuring the serum level of copper and different investigations of serum level of copper in different trimesters of pregnancy.

According to a descriptive study conducted by Navai et al. [36] in Tehran, deficiency in copper was significantly prevalent in nearly 60%. The study also noted that women were more likely than men to suffer from copper deficiency, and extensive research is needed to evaluate the prevalence of copper deficiency in different regions to take the required measures to compensate for it and achieve the ideal state of pregnancy as well as prevent the adverse maternal and fetal effects due to its deficiency. Deficiency of this micronutrient during pregnancy may lead to oxidative stress, resulting in reduced fetal growth [19]. The copper showed to play an important role in collagen and elastin production, and an insufficient level of this element can reduce the tensile strength of the amniotic sac, resulting in a rupture of the amniotic sac and preterm birth [37]. Of note is that most supplementations used during pregnancy do not contain copper; therefore, it is recommended that copper be added to supplementations during pregnancy. The maximum allowable intake of copper during pregnancy is 10 mg per day. The level of copper absorption depends to some extent on one’s diet. If one follows a poor diet and suffers from malnutrition, the digestion and absorption of copper can be negatively affected [19, 38]. The level of copper in each crop depends on the copper in soil [39].

As evidenced, it may be better to focus more on the copper element during pregnancy and the prominent role it can play in preventing preterm birth, and to plan for nutrition improvement for pregnant women, supplementations for mothers during pregnancy, and take measures to enrich the soil of regions.

## Conclusion

In light of the present findings, it can be concluded that changes in serum levels of zinc and copper in mothers with preterm delivery probably indicate the biological role of these elements in the pathogenesis of preterm delivery. It requires more extensive epidemiological studies. Moreover, it is recommended that in investigating mothers with lower serum levels of copper and zinc, some supplementations of these elements be prescribed and the chances of preterm delivery be compared with mothers with no consumption of any supplementation.

## Data Availability

The datasets used and/or analysed during the current study available from the corresponding author on reasonable request.
